# Influence of Temperature on Reaction Pathways in Fructose Conversion over Mixed Oxide Catalysts Based on Ce and Nb

**DOI:** 10.1021/acsomega.6c06462

**Published:** 2026-08-31

**Authors:** Dhara Beatriz de Amorim Pryston, Thatiane Veríssimo dos Santos Martins, Jailma Barros dos Santos, Daniel Santos Vilela Ferreira, Wemerson do Nascimento Silva, Renato da Silva Ferreira, Maria do Carmo M. Alves, Jonder Morais, Janaína Heberle Bortoluzzi, Mario Roberto Meneghetti, Simoni Margareti Plentz Meneghetti

**Affiliations:** † Group of Catalysis and Chemical Reactivity, Institute of Chemistry and Biotechnology, Federal University of Alagoas, Av. Lourival de Melo Mota, s/n°, 57072-970 Maceió, AL, Brazil; ‡ Instituto de Química, 28124 Universidade Federal do Rio Grande do Sul, CP 15003, 91501-970 Porto Alegre, RS, Brazil; § Electron Spectroscopy Lab, Instituto de Física, Universidade Federal do Rio Grande do Sul, CP 15051, 91501- 970 Porto Alegre, RS, Brazil

## Abstract

The influence of temperature on the product distribution during fructose conversion was systematically investigated using Ce­(P), Nb­(P), CeNb5­(P), CeNb15­(P), and CeNb25­(P) materials prepared by the Pechini method. Reactions were performed in aqueous medium for 2 h at temperatures ranging from 130 to 160 °C. Increasing temperature enhanced fructose conversion but also modified the relative contributions of dehydration, fragmentation, and consecutive degradation pathways. Nb­(P) reached the highest fructose conversion, increasing from 17.6% at 130 °C to 67.9% at 160 °C, but also exhibited a greater contribution from unidentified and degradation products under the most severe conditions. In contrast, the Ce–Nb mixed oxides, particularly CeNb15­(P) and CeNb25­(P), more effectively directed the identified soluble products toward 5-hydroxymethylfurfural (5-HMF), while limiting some competing pathways. The formation of C3 compounds, including dihydroxyacetone and pyruvaldehyde, indicates the occurrence of C–C bond cleavage reactions, whose contribution increased with temperature and depended on material composition. The observed product distributions result from the combined influence of temperature, acid-site nature, concentration, strength, and accessibility, together with the redox properties of the Ce containing materials. These findings demonstrate that the principal contribution of the studied oxides lies in their catalyst dependent modulation of the fructose reaction network rather than in the effect of temperature alone.

## Graphical abstract

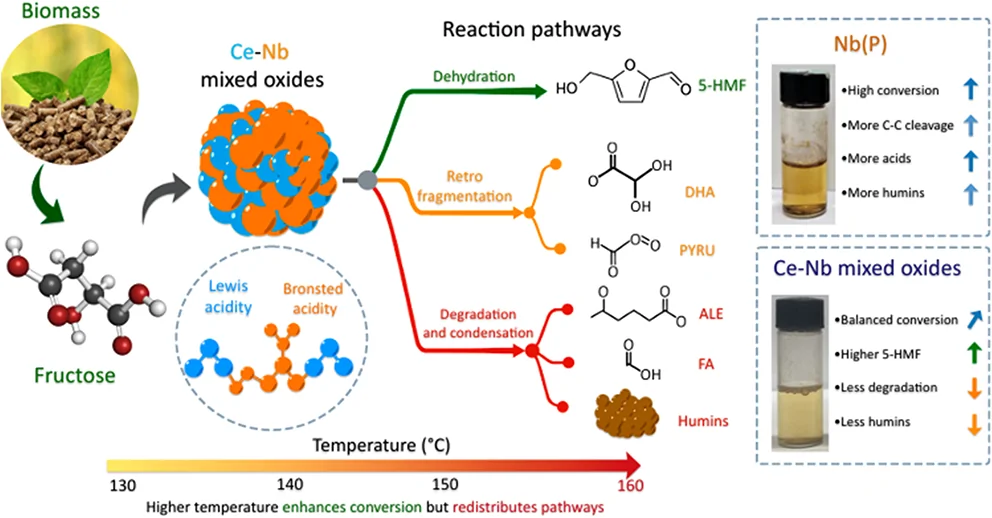

## 1 Introduction

The increasing demand for sustainable chemical processes has intensified the search for renewable carbon sources capable of replacing fossil-based feedstock.^1–4^ Among these alternatives, lignocellulosic biomass stands out due to its abundance, renewability, and potential to produce value-added chemicals. In this context, carbohydrates derived from biomass, particularly fructose, have attracted considerable attention as versatile platform molecules for the synthesis of a wide range of products, including 5-hydroxymethylfurfural (5-HMF).^5–9^

The conversion of fructose into 5-HMF typically proceeds via acid-catalyzed dehydration reactions.^10–13^ However, achieving high selectivity remains a major challenge due to the occurrence of parallel and consecutive pathways, which lead to the formation of byproducts such as organic acids, humins, and low molecular weight oxygenates.^14^ In addition to dehydration, fructose can undergo isomerization, retro-aldol fragmentation, and further degradation reactions, resulting in a complex reaction network strongly dependent on both catalyst properties and reaction conditions.^15,16^

Among the various parameters affecting fructose conversion, temperature plays a critical role in determining not only the reaction rate but also the distribution of products. While higher temperatures generally enhance conversion, they also tend to accelerate competing pathways, including C–C bond cleavage via retro-aldol reactions and the formation of degradation products.^10,17^ Despite its importance, the effect of temperature is often discussed primarily in terms of conversion or yield, with limited attention given to its impact on the overall reaction network and pathway redistribution.^10,17,18^

Heterogeneous catalysts based on transition metal oxides have been widely investigated for biomass conversion reactions.^19–21^ Niobium-containing materials possess both Brønsted and Lewis acid sites and can promote transformations such as isomerization and C–C bond cleavage.^11,22–24^ When combined with cerium oxide, which exhibits unique redox properties and oxygen mobility, Ce–Nb mixed oxides can provide a multifunctional catalytic surface capable of simultaneously promoting various reaction routes.^24–27^ However, the relationship between temperature and the catalytic properties of these systems, especially in terms of selectivity, remains insufficiently explored.

In this work, the influence of temperature on the product distribution in fructose conversion over a series of Ce–Nb oxide catalysts prepared by the Pechini method was investigated. By performing reactions at a fixed reaction time (2 h) and varying only the temperature, it is possible to directly assess how thermal effects influence the balance between dehydration, retro-aldol, and degradation pathways. With a comprehensive analysis of the reaction products, it is possible to identify distinct routes and to provide new insights into the role of temperature as a key parameter for controlling selectivity in biomass conversion.

Therefore, the main contribution of this study lies not in the general effect of temperature on reaction rates, but in demonstrating how Ce, Nb, and Ce–Nb based materials with distinct acidic and physicochemical properties modify the distribution of fructose conversion pathways under identical aqueous phase conditions. By correlating catalyst composition and acid site characteristics with dehydration, retro-aldol fragmentation, consecutive degradation, and the formation of unidentified products, this work provides insight into the catalyst dependent control of the fructose reaction network.

## 2 Experimental Section

### 2.1 Materials

Ammonium niobate­(V) oxalate hydrate (99.99%, trace metals basis) and cerium­(III) nitrate hexahydrate (99.999%, trace metals basis) were purchased from Sigma-Aldrich. Citric acid (>99.0%) and glycerol (>99.0%) were also obtained from Sigma-Aldrich. Ammonium hydroxide solution (28–30% NH_3_) and glacial acetic acid (≥99.7%) were supplied by Dinâmica. Chitosan (medium molecular weight) was acquired from Sigma-Aldrich. Fructose (>99.0%) and sulfuric acid (>95%) were commercially available and used without further purification. Deionized water was employed throughout all experiments.

### 2.2 Catalyst Synthesis and Characterization

Details concerning catalyst synthesis and physicochemical characterization are available in our previous work^28^ and presented in the Supporting Information. The designations of the prepared materials are Ce­(P) and Nb­(P) for pristine cerium oxide and niobium oxide, respectively. Mixed oxide containing 5%, 15% and 25% (wt %) of niobium are designed as CeNb5­(P), CeNb15­(P), CeNb25­(P), respectively.

Briefly, the materials were prepared by a modified Pechini method using cerium nitrate and/or ammonium niobate oxalate as metal precursors, citric acid as a complexing agent, and glycerol as the polyol. After resin formation, the solids were subjected to an initial thermal treatment, ground and sieved, and subsequently calcined at 550 °C for 4 h. Full synthesis and characterization details are provided in the Supporting Information.

### 2.3 Catalytic Tests

Catalytic performance was evaluated in fructose conversion reactions performed in sealed glass vials under magnetic stirring. Experiments were carried out at temperatures ranging from 130, 140, 150, and 160 °C with a fixed reaction time of 2 h, using 0.016 g of fructose in 2.0 mL of deionized water and 0.0030 g of catalyst. It is important to mention that (i) this fructose concentration and catalyst loading were selected based on preliminary experiments and previous studies to ensure measurable conversion, adequate solid substrate contact, and reliable HPLC analysis while minimizing humin formation, and (ii) the catalytic results are reported as apparent conversions (%) and yields (%) under the applied experimental conditions, since dedicated tests to exclude internal and external mass-transfer limitations were not performed. After reaction, the mixtures were filtered through 0.45 μm membrane filters (Millipore) prior to analysis. The liquid phase was analyzed by high-performance liquid chromatography (HPLC) equipped with a refractive index detector (Shimadzu CTO-20A system) using a Varian MetaCarb H Plus column (300 × 7.8 mm) and a corresponding guard column. Detailed operating conditions are provided in the Supporting Information. Quantification was performed using calibration curves obtained from standard solutions, and fructose conversion, product yield, and selectivity were calculated based on concentration data, as detailed in the Supporting Information. Considering the combined contributions from sample preparation, injection repeatability, calibration, and chromatographic integration, the overall experimental error associated with the chromatographic determinations was estimated to be approximately 5%.

Reusability tests were performed using CeNb25­(P) at 160 °C for 2 h under the same reaction conditions employed in the catalytic experiments. After each cycle, the catalyst was recovered by centrifugation, calcined at 550 °C for 4 h, and reused. This procedure was repeated for four consecutive cycles. The liquid phases collected after each cycle were analyzed to assess potential leaching of Ce and Nb.

## 3 Results and Discussion

The influence of temperature on fructose conversion was systematically investigated at 130, 140, 150, and 160 °C, at 2 h of reaction, using Ce­(P), Nb­(P), CeNb5­(P), CeNb15­(P), and CeNb25­(P) catalysts prepared via the Pechini method. As temperature can directly affect both catalytic activity and the extent of secondary reactions, its evaluation is essential to understand the interrelation between conversion, selectivity, and humin formation in these systems.

Initially, the physicochemical properties of the catalysts synthesized by Pechini method, summarized in Table 1, reveal a strong dependence on niobium incorporation, affecting both surface acidity and composition.^26^ The total acidity increased markedly with Nb content, from 318 mmol NH_3_ g^–1^ for Ce­(P) to 830 and 931 mmol NH_3_ g^–1^ for CeNb25­(P) and CeNb15­(P), respectively, accompanied by a progressive increase in the fraction of strong acid sites, reaching up to 56.5% for CeNb25­(P). The quantitative analysis of pyridine adsorption showed that Brønsted acid sites predominate in all materials, with B/L ratios ranging from 1.15 for Ce­(P) to 3.27 for Nb­(P). The FTIR spectra of adsorbed pyridine and the corresponding concentrations of Brønsted and Lewis acid sites are presented in Figure S1 of the Supporting Information. The concentrations were calculated from the integrated areas of the characteristic bands using the respective extinction coefficients, as described in the Supporting Information

**Table 1:** Results of Texturalproperties, Mean Particle Size (D̅), Polydispersity Index­(PI), and Amount of Acid and Basic Sites for the Materials

	Textural Properties					Acid sites^f^					Elements (%)		
Sample	S_BET_ ^a^(m^2^g^–1^)	Vp^b^(cm^3^g^–1^)	Dp_BJH_ ^c^(Å)	D̅^d^(nm)	PI^e^	Total (mmol NH_3_ g^–1^)	Weak (%)	Medium (%)	Strong (%)	B/L ratio^g^	XPS		EDX
Ce(P)	70.6	0.161	36.20	686	0.38	318	27.6	45.8	26.6	1.15	Ce	12	-
											O	88	-
Nb(P)	48.5	0.127	79.07	361	0.74	433	2.1	53.6	44.3	3.27	Nb	15	-
											O	85	-
CeNb5(P)	95.2	0.181	36.00	326	0,70	410	9.9	58.0	32.0	1.34	Ce	11	-
											Nb	2	3.8
											O	87	-
CeNb15(P)	55.4	0.156	35.93	464	0,77	931	7.3	45.1	47.7	2.07	Ce	9	-
											Nb	5	11.3
											O	86	-
CeNb25(P)	56.1	0.100	36.22	507	0,71	830	4.4	39.1	56.5	3.26	Ce	7	-
											Nb	8	18.8
											O	85	-

a *S*_BET_, BET specific surface area.

b *Vp*, pore volume.

c *Dp*_BJH_, pore diameter.

d D̅, mean size.

e PI = polydispersity index.

f Total acidity and acid strength distribution determined by NH_3_-TPD; B/L ratio determined by FTIR spectroscopy of adsorbed pyridine. The corresponding spectra and quantitative Brønsted and Lewis acid-site concentrations are presented in Figure S1. The specific surface areas, total pore volumes, and pore diameters were calculated by the BET and BJH equations.

XPS results suggest the progressive surface enrichment in Nb, with Nb content increasing from 2% in CeNb5­(P) to 8% in CeNb25­(P), while Ce content decreased accordingly, indicating preferential exposure of Nb species at the surface. Additionally, the presence of Ce^4+^/Ce^3+^ species implies the existence of oxygen vacancies, which may contribute to enhanced surface reactivity.

These findings demonstrate that the Pechini method enables fine-tuning of the surface properties of Ce–Nb oxides, particularly in terms of acidity and electronic structure, which are key parameters governing their catalytic behavior.^17^

The FTIR spectra of adsorbed pyridine and the quantitative distribution of Brønsted and Lewis acid sites are presented in Figures S1. The B/L ratios reported in Table 1 were higher than unity for all materials, indicating that the concentration of Brønsted acid sites was greater than that of Lewis acid sites. Ce­(P) exhibited the lowest B/L ratio (1.15), corresponding to a relatively balanced distribution of the two types of acid sites. In contrast, CeNb25­(P) showed a substantially higher B/L ratio (3.26), very similar to that observed for Nb­(P) (3.27). The quantitative data indicate that, for CeNb25­(P), this higher B/L ratio is mainly associated with a greater concentration of Brønsted acid sites, while the concentration of Lewis acid sites remains similar to that observed for the other Nb containing materials. It should be emphasized that the B/L ratio reflects the relative concentrations of Brønsted and Lewis acid sites and does not provide information about their intrinsic acid strength.

The higher concentration of Brønsted acid sites in CeNb25­(P) may be associated with proton donating surface hydroxyl groups, including Ce–OH and Nb–OH species, as well as hydroxylated environments related to Ce–O–Nb linkages. Under aqueous reaction conditions, coordinated water molecules may also become polarized and contribute to proton donating surface species.

The overall acid-strength distribution was evaluated by NH_3_-TPD. The results indicate that Ce­(P) contains a greater contribution from weak-to-medium acid sites, whereas CeNb25­(P) exhibits a greater contribution from medium-to-strong acid sites. However, NH_3_-TPD does not directly distinguish between Brønsted and Lewis sites. Therefore, although pyridine-FTIR shows a higher concentration of Brønsted than Lewis sites in CeNb25­(P), the available data do not demonstrate that the Brønsted sites are intrinsically stronger.

Figure 1 presents the fructose conversion profiles obtained for the different catalytic systems at temperatures ranging from 130 to 160 °C. For all catalysts, fructose conversion increased markedly with temperature, confirming the strong thermal dependence of the reaction.^22,29–31^ Among the evaluated catalysts, Nb­(P) exhibited superior performance across the entire temperature range, reaching the greatest fructose conversion at 160 °C. This behavior reflects the higher apparent performance under the investigated conditions of Nb-containing catalysts toward fructose conversion.^32–34^ Mixed oxides such as CeNb15­(P) and CeNb25­(P) also exhibited substantial conversion, particularly at elevated temperatures, demonstrating that Ce incorporation does not suppress catalytic activity. Considering the physicochemical properties summarized in Table 1, the catalytic performance appears to be governed primarily by the distribution and strength of the acid sites rather than by variations in textural properties.

**Figure 1:**
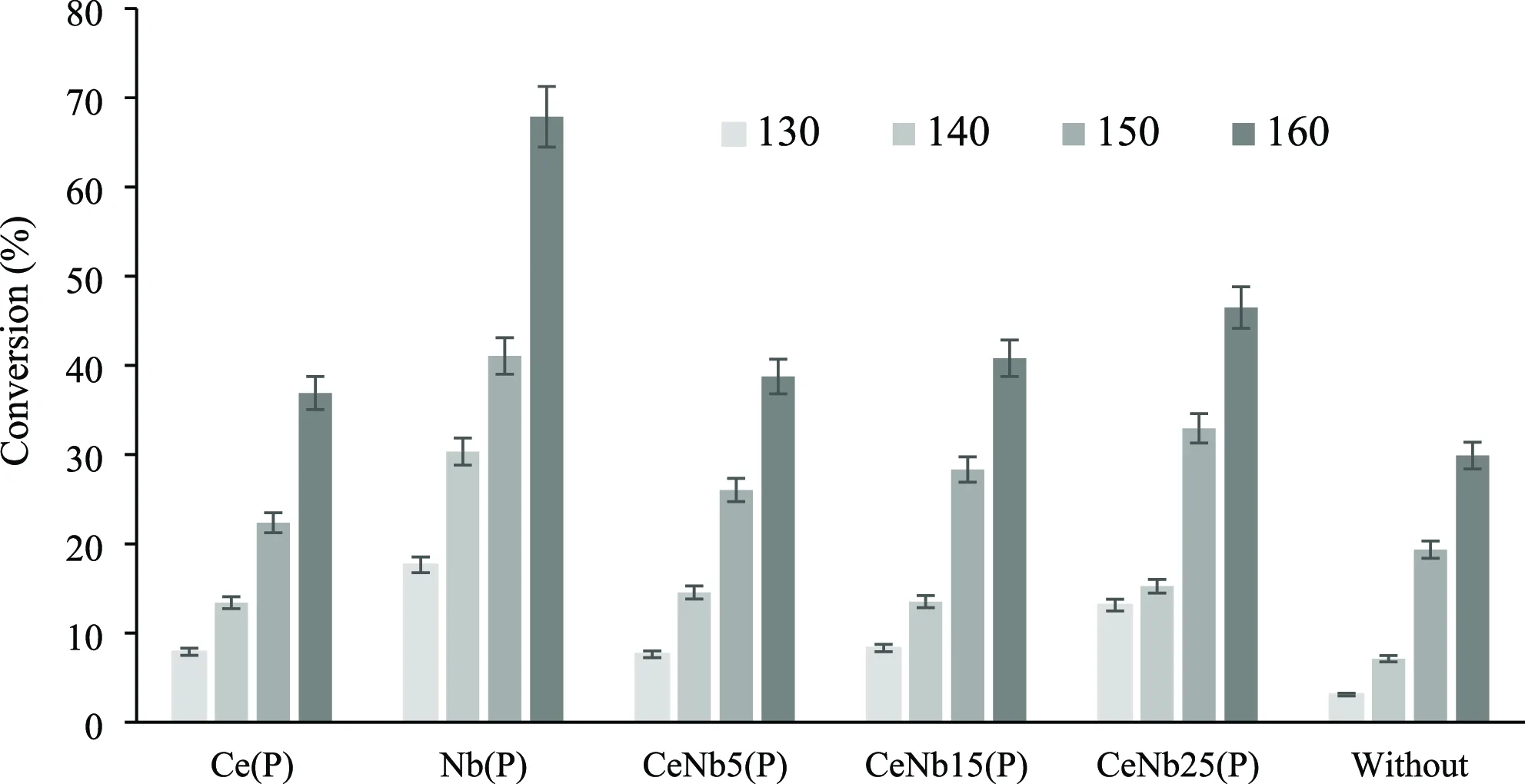
Effect of temperature on fructose conversion (%) using Ce­(P), Nb­(P), CeNb5­(P), CeNb15­(P) and CeNb25­(P) at different temperatures (130, 140, 150, and 160 °C, at 2 h of reaction) (error bars represent the estimated relative experimental uncertainty of ± 5% associated with the chromatographic determinations).

Even in the absence of a catalyst, fructose conversion occurred, particularly at higher temperatures, demonstrating the contribution of thermal effects to the reaction, although the markedly higher conversions obtained in the presence of catalysts confirm their fundamental role in promoting fructose transformation.

Regarding the formation of soluble products (Table S1 and Figure 2), several compounds were identified, including glucose (G), 5-hydroxymethylfurfural (5-HMF), glyceraldehyde (GLY), pyruvaldehyde (PYRU), dihydroxyacetone (DHA), as well as organic acids such as levulinic acid (LA), formic acid (FA), and acetic acid (AA). It is important to highlight that mannose may also be formed through fructose isomerization,^12^ but its formation could not be reliably identified or quantified in the present study.

**Figure 2:**
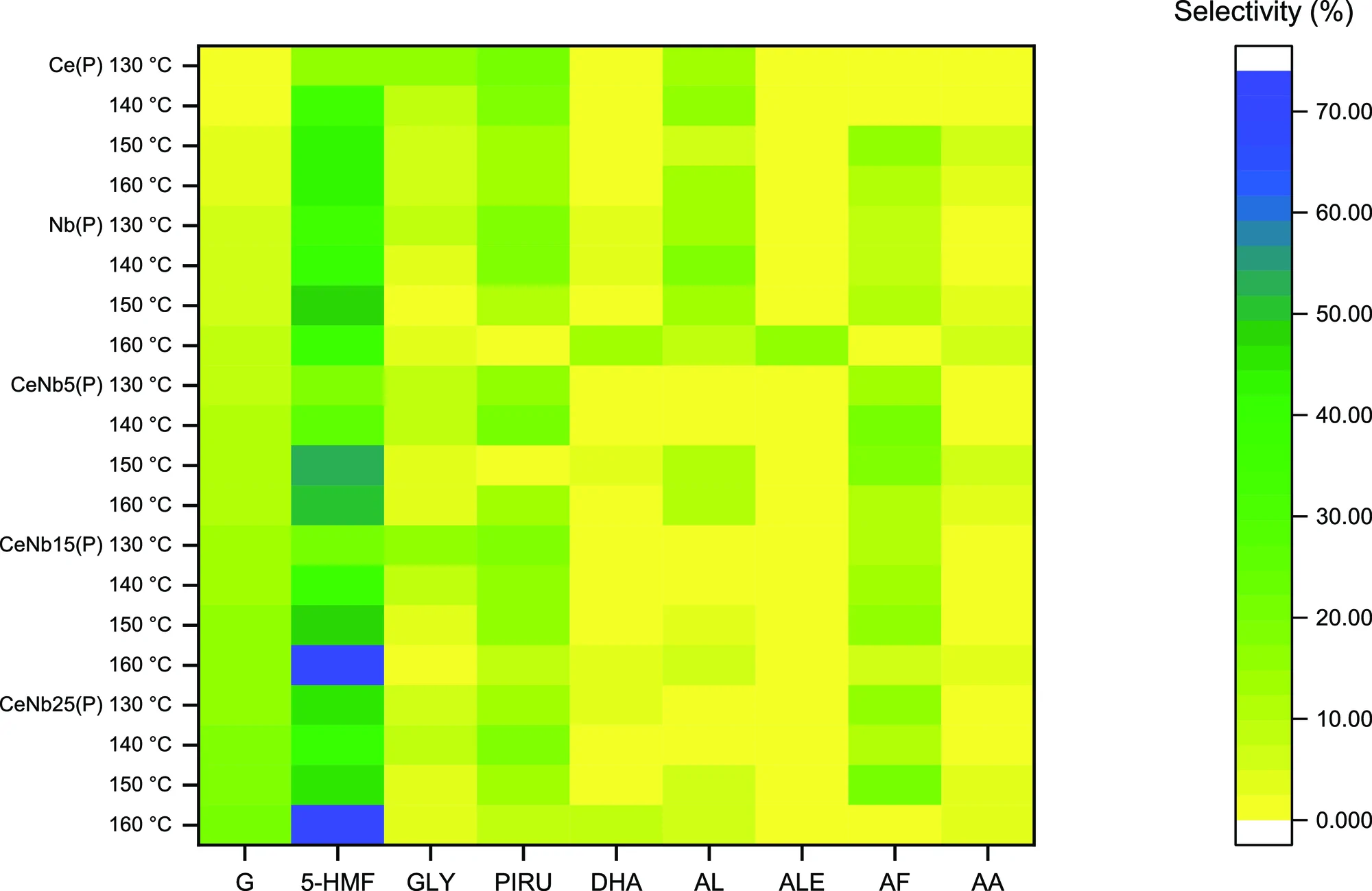
Temperature-dependent distribution of soluble products obtained from fructose conversion using Ce­(P), Nb­(P), CeNb5­(P), CeNb15­(P) and CeNb25­(P) at different temperatures (130, 140, 150, and 160 °C, at 2 h of reaction). Glucose (G), 5-hydroxymethylfurfural (5-HMF), glyceraldehyde (GLY), pyruvaldehyde (PIRU), dihydroxyacetone (DHA), levulinic acid (LA), formic acid (FA), and acetic acid (AA); results expressed as selectivity (%).

Regarding the product distribution, increasing the reaction temperature generally favors fructose dehydration across all catalytic systems, reflecting the thermally activated nature of the reaction. However, the extent of this effect strongly depended on catalyst composition. While Ce­(P) exhibited only a moderate increase in dehydration products with temperature, Nb-containing catalysts, particularly CeNb15­(P) and CeNb25­(P), showed a much more pronounced response, reaching the highest selectivity toward 5-HMF at 160 °C. These results suggest that Nb incorporation enhances the efficiency of fructose dehydration under elevated temperatures.

The appreciable 5-HMF selectivity observed at the lowest measured fructose conversions suggests its direct formation through catalyst assisted dehydration (Figure S2, Supporting Information). Still, because the available data set does not contain sufficient points near zero conversion, 5-HMF cannot be conclusively classified as a primary product over the Ce–Nb oxides, and future experiments at shorter reaction times and lower conversions will enable a rigorous delplot analysis.^12^

Still, the selectivity versus conversion plots obtained at 160 °C (Figure S2, Supporting Information). also allow the catalysts to be compared at similar fructose conversion levels under identical thermal conditions. At comparable conversions, the Ce–Nb mixed oxide exhibited higher selectivity toward 5-HMF than pristine Nb­(P), whereas Nb­(P) showed a greater contribution from fragmentation and consecutive degradation products. This behavior can be associated with the different apparent activation energies of the parallel and consecutive reactions involved in fructose conversion. Kinetic studies have shown that both fructose dehydration to 5-HMF and competing degradation pathways are strongly temperature dependent, so increasing temperature may accelerate not only the desired reaction but also fragmentation, 5-HMF rehydration, and humin formation.^35,36^ Since 5-HMF is an intermediate product, its observed yield results from the balance between its formation from fructose and its subsequent consumption through rehydration, condensation, and degradation pathways.^36,37^

These differences demonstrate that product distribution is governed not only by the conversion level, but also by catalyst composition and by the nature, strength, and accessibility of the acid sites.

In addition to the formation of 5-HMF, the effect of temperature on intermediate species formation provides important insights into the progression of the reaction network. Compounds such as GLY and G, which are associated with initial transformation steps of fructose, showed a consistent decrease in concentration as the temperature increased. This performance suggests that these species are not final products but rather transient intermediates that are progressively consumed under more severe reaction conditions. The decrease in their concentration with increasing temperature indicates that higher thermal energy accelerates subsequent reaction steps, driving the system toward more stable products such as 5-HMF or toward fragmentation and degradation pathways.^12,38^

Notably, this trend was observed across all catalysts, although the rate of consumption varied depending on the catalyst composition. Nb containing systems exhibited a faster consumption of these intermediates, indicating higher overall reactivity. This behavior may be associated with their higher overall acidity and with the combined contribution of Brønsted and Lewis acid sites, rather than with Lewis acidity alone. These results reinforce the idea that temperature plays a central role in controlling not only product formation but also the lifetime and transformation of key intermediates in the reaction network.

Further insights into the activation of fragmentation pathways were obtained from the formation of C3 compounds such as DHA and PIRU, typically associated with retro-aldol reactions during fructose conversion.^39^ Higher temperatures promoted the formation of these products, particularly in Nb containing catalysts, indicating that more severe reaction conditions favor the generation of smaller oxygenated molecules. This behavior also suggests an important role of Nb species in promoting retro-aldol transformations.^40^

It is worth noting that intermediates such as GLY, which are expected to be formed during retro-aldol reactions, were not directly detected. However, the simultaneous presence of DHA and PYRU strongly suggests that such intermediates are formed transiently and rapidly converted into more stable products under the reaction conditions. These findings indicate that increasing temperature promotes a shift in the reaction network from selective dehydration toward fragmentation and consecutive degradation pathways, particularly over the more acidic Nb containing materials. This behavior likely results from the combined effects of acid site nature, concentration, strength, and accessibility, rather than from Lewis acidity alone.^41^

Nevertheless, the formation of products such as AL, AF, ALE, and AA provides important information about the occurrence of consecutive reactions and degradation processes. These compounds are typically associated with secondary transformations, either from 5-HMF or from smaller intermediates formed via fragmentation pathways. As the temperature increased, a clear rise in the concentration of these products was observed, indicating that higher temperatures not only promote primary reactions but also accelerate subsequent transformations. In particular, the formation of AA can be considered a strong indicator of deeper degradation, reflecting more severe reaction conditions. The extent of these processes was found to depend on the catalyst composition, since CeNb15­(P) and CeNb25­(P) maintained relatively high 5-HMF yields even at elevated temperatures, although Nb­(P) showed a more pronounced formation of degradation products, suggesting that its higher overall acidity and greater contribution of medium-to-strong acid sites, combined with high temperature, may favor undesired pathways. These results highlight the relationship between activity and selectivity, demonstrating that although higher temperatures enhance conversion, they also increase the possibility of product degradation, especially in highly reactive catalytic systems.

In this context, Figure 3 presents the visual appearance of the reaction media after fructose conversion, providing qualitative evidence of humin formation as a function of catalyst composition. A pronounced darkening of the solution was observed for Nb­(P), whereas Ce­(P) and the mixed oxides exhibited considerably lighter reaction media. These differences suggest variations in the extent of insoluble product formation depending on the catalyst composition.

**Figure 3:**
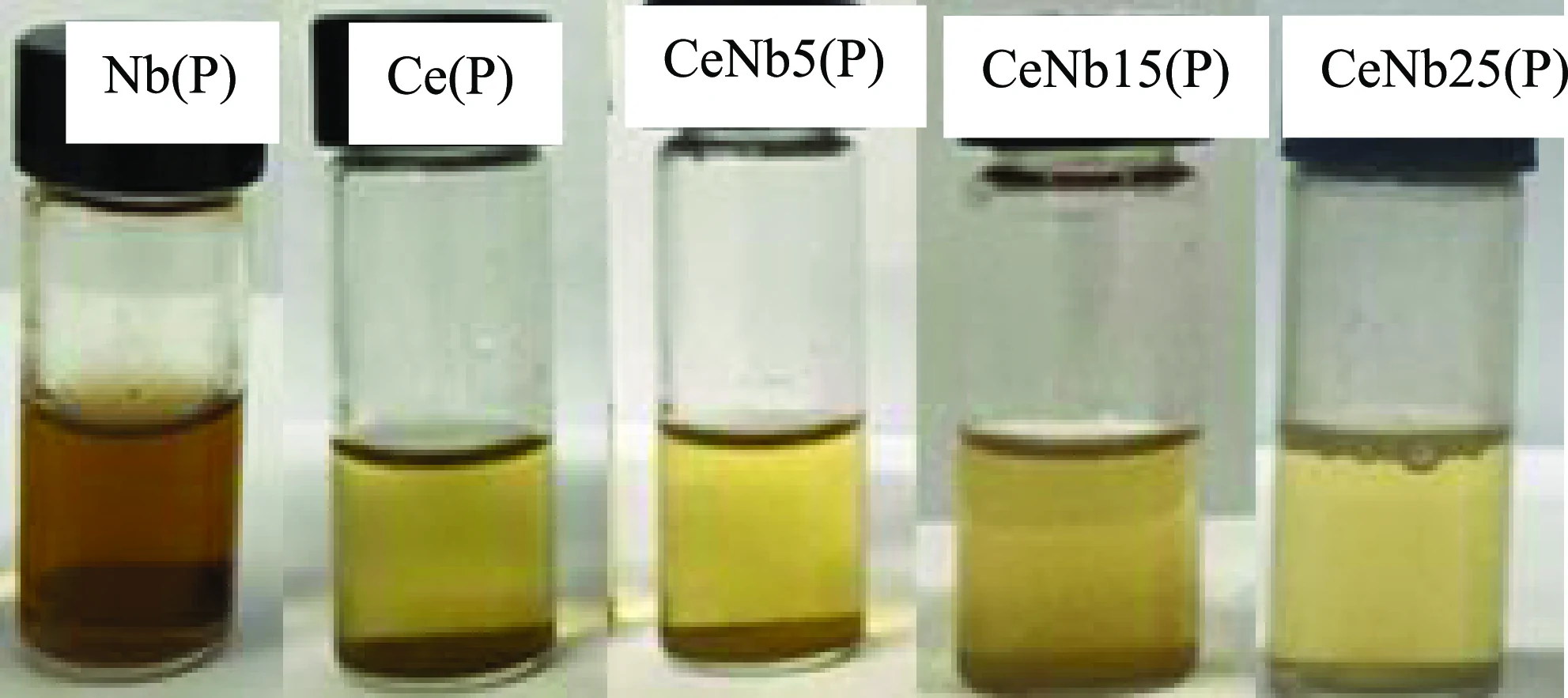
Visual aspect of reaction medium after fructose conversion in the presence of different catalysts: Nb­(P), Ce­(P), CeNb5­(P), CeNb15­(P), and CeNb25­(P), at 160 °C and 2 h of reaction.

The darker reaction medium observed for Nb­(P) is consistent with its high catalytic activity and suggests a greater extent of humin formation and other insoluble condensation products under severe reaction conditions.^42^ In contrast, reactions carried out in the presence of Ce­(P) resulted in considerably lighter media, suggesting lower humin formation, although accompanied by reduced fructose conversion. Reactions performed with the mixed oxides exhibited intermediate behavior, maintaining appreciable catalytic activity while minimizing excessive darkening of the reaction medium. These results suggest that Ce incorporation contributes to moderating secondary condensation reactions, leading to a more balanced catalytic performance.^43–46^

The visual differences observed in Figure 3 motivated a more detailed evaluation of the relationship between fructose conversion and product distribution. For this purpose, Figure 4 compares fructose conversion with the fraction of soluble products identified in the liquid phase at 160 °C (see also Figure S3), since increased conversion was not always accompanied by a proportional recovery of quantified soluble products (Table S1). The comparison between total fructose conversion (Figure 1) and the sum of the identified soluble product yields (Table S1) reveals the presence of an unidentified fraction, particularly under the more severe reaction conditions. The reactions performed at 130, 140, and 150 °C (Figure S4) showed the same general trend observed at 160 °C.

**Figure 4:**
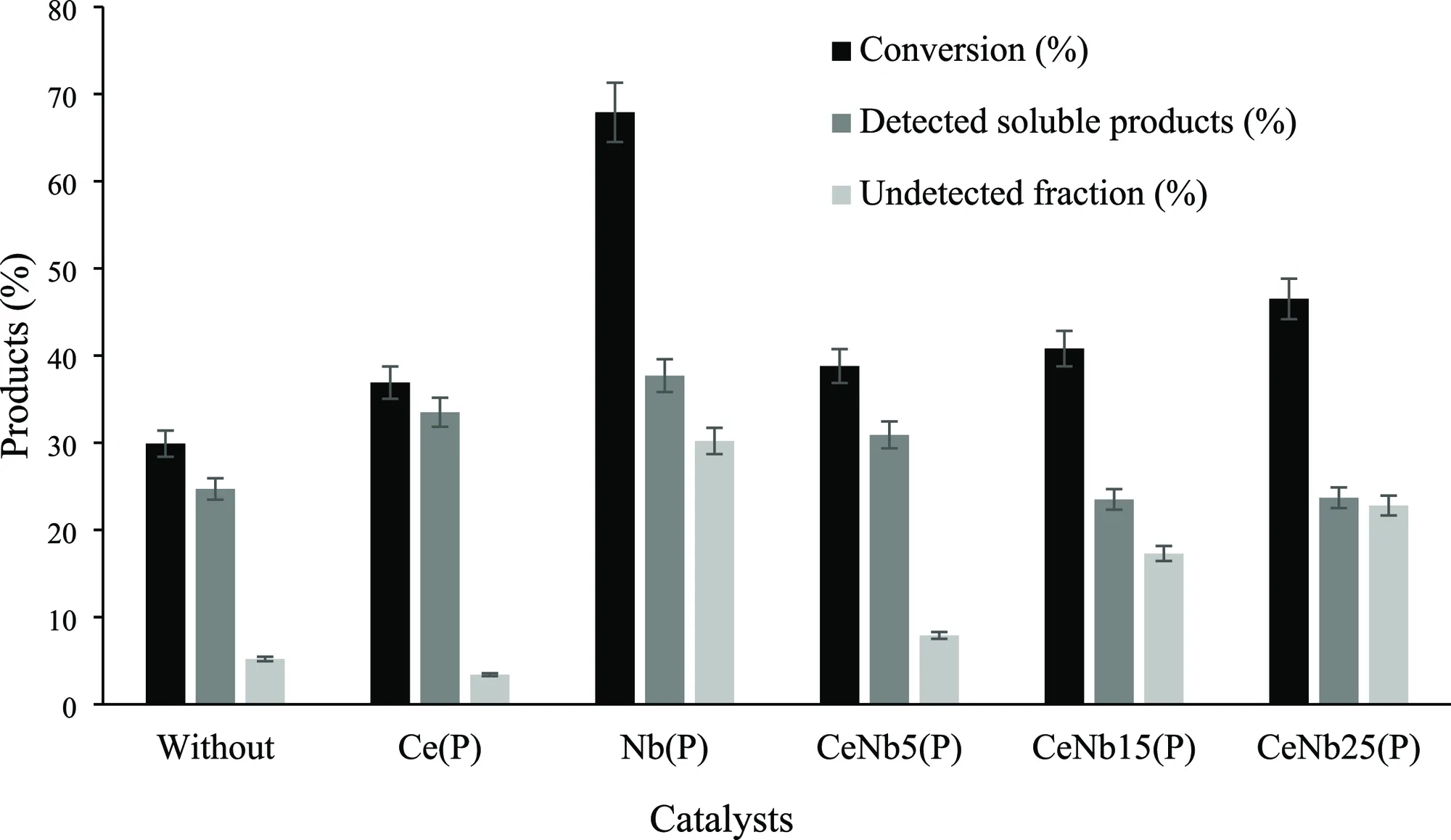
Comparison between fructose conversion (%) at 160 °C, detected soluble products (%) and unidentified fraction (%) for Ce–Nb catalysts (Unidentified fraction (%) = Conversion (%)—soluble products identified (%)); error bars represent the estimated relative experimental uncertainty of ± 5% associated with the chromatographic determinations.

Because TOC measurements were not available and the gas phase was not analyzed, complete mass and carbon balances could not be established. Therefore, the difference between fructose conversion and the sum of the identified soluble product yields is reported as an unidentified fraction. This fraction may comprise unidentified soluble compounds, volatile products, insoluble condensation products, and a contribution from the approximately 5% experimental uncertainty associated with the chromatographic determinations. Accordingly, it should not be interpreted exclusively as humin formation.

The product distribution profiles shown in Figure 2 indicate that increasing the reaction temperature promotes not only fructose dehydration but also competing fragmentation and consecutive degradation pathways. Under these conditions, reactive intermediates such as 5-HMF may undergo secondary rehydration, condensation, and polymerization reactions, contributing to the formation of insoluble species and decreasing the recovery of identified soluble products (see Table S1).

Among the evaluated materials, Nb­(P), which exhibited the highest fructose conversion at 160 °C, also showed the largest difference between total conversion and the yields of the identified products. This behavior, together with the pronounced darkening of the reaction medium observed in Figure 3, suggests extensive formation of humins and other unidentified products. Thus, although Nb­(P) promoted the greatest fructose conversion, a substantial fraction of the converted substrate was directed toward undesired secondary pathways.

In contrast, Ce­(P) showed a considerably smaller unidentified fraction, suggesting a lower contribution from unidentified secondary pathways (Figure 1). The mixed oxides provided an intermediate and composition-dependent response. CeNb5­(P) showed a favorable balance between fructose conversion and recovery of identified soluble products, whereas CeNb15­(P) and CeNb25­(P) more effectively directed the reaction toward 5-HMF while limiting the contribution of some undesired pathways. These results indicate that the incorporation of cerium modulates the physicochemical properties of the Nb containing materials, reducing excessive secondary reactions without completely suppressing fructose conversion.

The temperature dependent redistribution of the reaction pathways can be rationalized based on the nature, relative concentration, strength, and accessibility of the acid sites. Pyridine-FTIR analysis indicated a predominance of Brønsted over Lewis acid sites, particularly for CeNb25­(P), whereas NH_3_-TPD showed a greater contribution of medium-to-strong acid sites for the Nb containing materials. Brønsted acid sites are mainly associated with proton assisted fructose dehydration to 5-HMF, but they may also contribute to subsequent rehydration, condensation, and degradation reactions. Lewis acid sites may coordinate and polarize oxygen containing functional groups, facilitating ring opening, rearrangement, isomerization, and C–C bond-cleavage steps. Therefore, the formation of C3 products such as DHA and PYRU should not be attributed exclusively to Lewis acidity, but rather to the combined action of the different acid sites under the applied reaction conditions.

The contribution of cerium must also be considered, since the Ce^4+^/Ce^3+^ redox pair and the associated oxygen vacancies may modify the electronic environment of Nb species, influence substrate adsorption, and affect the stability and transformation of reaction intermediates. Consequently, the behavior of the mixed oxides results from the interplay among acidity, redox properties, and catalyst composition.

Reviewing, the results demonstrate that temperature alone does not determine the product distribution. Its effect is modulated by the composition of the material and by nature, relative concentration, strength, and accessibility of the acid sites. At higher temperatures, the high overall acidity and substantial contribution of medium-to-strong acid sites of Nb­(P) favor a broader range of dehydration, fragmentation, and degradation reactions. In comparison, the tuned acid and redox properties of the Ce–Nb mixed oxides, especially CeNb15­(P) and CeNb25­(P), favor 5-HMF formation while restricting some consecutive degradation pathways. Thus, catalyst performance should be evaluated by considering fructose conversion together with product selectivity and the recovery of identified soluble products, rather than conversion alone.^35–37^

The stability and reusability of CeNb25­(P) were evaluated over four consecutive reaction cycles at 160 °C for 2 h. After each cycle, the material was recovered by centrifugation, calcined at 550 °C for 4 h, and reused under the same reaction conditions. As shown in Figure S5A, fructose conversion remained essentially constant throughout the four consecutive cycles, ranging from 45.1 to 46.8%, with no progressive loss of performance. In addition, the XRD pattern recorded after the fourth cycle showed that the recovered material remained crystalline, supporting its structural stability under the investigated conditions. These results suggest that CeNb25­(P) exhibits good structural stability and can be effectively recovered and reused under the investigated conditions.

## 4 Conclusions

The influence of temperature on fructose conversion over Ce­(P), Nb­(P), CeNb5­(P), CeNb15­(P), and CeNb25­(P) materials prepared by the Pechini method was systematically investigated, with emphasis on product selectivity and reaction pathway distribution. The main contribution of this work is to demonstrate that Ce, Nb, and Ce–Nb based oxides modify the fructose reaction network differently according to their composition and physicochemical properties. Therefore, the reaction outcome is determined not only by temperature, but also by the material-dependent balance among dehydration, fragmentation, and consecutive degradation pathways.

Fructose conversion increased with temperature for all the evaluated systems. Nb­(P) reached the highest conversion throughout the investigated temperature range; however, it also exhibited the largest unidentified fraction and a greater contribution from fragmentation and degradation products under the most severe conditions. Although the pronounced darkening of the reaction medium is consistent with the formation of humins and other condensation products, the unidentified fraction cannot be assigned exclusively to insoluble products because TOC measurements and gas-phase analyses were not performed.

The Ce–Nb mixed oxides displayed a more balanced and composition-dependent behavior. CeNb5­(P) combined moderate fructose conversion with relatively high recovery of identified soluble products, whereas CeNb15­(P) and CeNb25­(P) more effectively directed the reaction toward 5-HMF while limiting some undesired secondary pathways. These differences indicate that product distribution is governed by the combined influence of acid-site nature, relative concentration, strength, and accessibility, together with the redox properties associated with Ce species and oxygen vacancies.

The appreciable 5-HMF selectivity observed at the lowest measured conversions is consistent with its direct formation through fructose dehydration. Nevertheless, the available data do not include sufficient points close to zero conversion to classify 5-HMF conclusively as a primary product, and experiments at shorter reaction times would be required for a rigorous delplot analysis. In addition, CeNb25­(P) maintained essentially constant fructose conversion over four consecutive cycles, supporting its reusability under the investigated conditions. Overall, the results demonstrate that tuning the composition and surface properties of Ce–Nb oxides provides an effective strategy for controlling the balance between fructose dehydration and competing reaction pathways.

## Supplementary Material



## Data Availability

All relevant data are available within the article and its Supporting Information.

## Notes

Are presented the preparation and characterization of catalysts synthesized by the modified Pechini method, Figure S1 presents the Pyridine-FTIR spectra for Ce­(P), Nb­(P), CeNb5­(P), CeNb15­(P), and CeNb25­(P), showing the bands assigned to Brønsted (B), Lewis (L), and combined Brønsted-Lewis (B + L) acid sites (A), and corresponding amounts of Brønsted and Lewis acid sites determined from the pyridine adsorption measurements (B), Figure S2 shows Selectivity of 5-HMF as a function of fructose conversion at 160 °C using Ce­(P), Nb­(P), CeNb5­(P), CeNb15­(P), and CeNb25­(P), Figure S3 presents Relationship between fructose conversion (%) and the formation of non soluble products (%), at 160 °C, Figure S4 shows Comparison between fructose conversion (%) at 130, 140, 150, and 160 °C, detected soluble products (%) and unidentified fraction (%) for Ce–Nb catalysts (Unidentified fraction (%) = Conversion (%)—soluble products identified (%)), Figure S5 presents Reusability test of CeNb25­(P) at 160 °C for 2 h, four consecutive cycles (A) and XRD pattern of CeNb25­(P) after the reuse tests (B) and Table S1 presents the soluble products identified in the fructose conversion at 130 °C, 140 °C, 150 and 160 °C, 2 h, without or using 3.0 × 10^–3^ g of Ce­(P), Nb­(P), CeNb5­(P), CeNb15­(P), and CeNb25­(P) (Yield (%)) (PDF)
