# Supplementary material for: Influence of Temperature on Reaction Pathways in Fructose Conversion over Mixed Oxide Catalysts Based on Ce and Nb
Source: ACS Omega. 2026 Aug 31;11(36):54492–500. doi: 10.1021/acsomega.6c06462 (PMC13584797; doi:10.1021/acsomega.6c06462)
Supplement: Supplementary file 1 [file ao6c06462_si_001.pdf]

Influence of temperature on reaction pathways in fructose conversion over mixed oxide catalysts based on Ce and Nb

Dhara Beatriz de Amorim Pryston[1], Thatiane Veríssimo dos Santos Martins[1], Jailma Barros dos Santos[1], Daniel Santos Vilela Ferreira[1], Wemerson do Nascimento Silva[1], Renato da Silva Ferreira[1], Maria do Carmo M. Alves[2], Jonder Morais[3], Janaína Heberle Bortoluzzi[1], Mario Roberto Meneghetti[1], Simoni Margareti Plentz Meneghetti[1]

[1] Group of Catalysis and Chemical Reactivity, Institute of Chemistry and Biotechnology, Federal University of Alagoas, Av. Lourival de Melo Mota, s/ n°, Maceió-AL – 57072-970, Brazil*simoni.plentz@gmail.com

[2] Instituto de Química, Universidade Federal do Rio Grande do Sul, CP 15003, 91501-970, Porto Alegre, RS, Brazil

[3] Electron Spectroscopy Lab, Instituto de Física, Universidade Federal do Rio Grande do Sul, CP 15051, 91501- 970, Porto Alegre, RS, Brazil

Supplementary material

Preparation and characterization of catalysts by the modified Pechini method

The synthesis began by preparing aqueous solutions of cerium nitrate hexahydrate ($Ce(NO_3)_3 \cdot 6H_2O$) and/or ammonium niobate(V) oxalate hydrate ($NH_4[NbO(C_2O_4)_2] \cdot xH_2O$) with citric acid (molar ratio 3:1). This excess promotes complete metal chelation through entropically driven polydentate

coordination, where each citrate ligand replaces multiple monodentate ligands, increasing the entropy of the system and favoring stable intermediate structures. The ratio prevents phase segregation during heat treatment [43]. In the subsequent step, both solutions were mixed at 70 °C for 30 minutes to form metal citrate. For mixed oxides, it was necessary to previously homogenize the niobium and cerium precursor solutions for 10 minutes at 30 °C, followed by simultaneous insertion into the citric acid solution, respecting variations in the mass proportion of cerium and niobium ($CeNb_x$, where x = 5, 15 and 25%). Subsequently, glycerol was added to the metal citrate solution at a molar ratio of 60:40 relative to the amount of citric acid. The solution was vigorously stirred at 70 °C for 2 hours to ensure the homogeneity of the system. Then, the temperature was increased to 130 °C to promote the polyesterification reaction and subsequent formation of the polymeric resin. Finally, the oxides were subjected to two calcination steps. Initially, the first stage occurred at 280 °C (for cerium oxide and mixed oxides) or 350 °C (for niobium oxide). After this step, the materials were macerated and sieved through 100 mesh sieves. Subsequently, all catalysts were subjected to a second calcination step at 550 °C for 4 hours.

X-ray diffraction (XRD) measurements were conducted using a Shimadzu diffractometer, model XRD-6000, with Cu Kα radiation (1.5418 Å) and a nickel filter, obtaining wide-angle diffraction patterns in the 2θ region of 3° at 40°. The crystallite size (nm) was calculated using the Debye–Scherrer equation. Nitrogen physisorption measurements were performed at 77.15 K with a gas adsorption analyzer (Micromeritics, model ASAP-2020). The textural properties were estimated from N_2 adsorption isotherms using the Brunauer–Emmett–Teller (BET) equation and the Barrett–Joyner–Halenda (BJH) method. Before analysis, all the samples were pretreated under vacuum at 423.15 K for 24 hours. The Temperature-Programmed Desorption (TPD-NH_3 and TPD-CO_2) measurements were performed using a Termolab multipurpose analytical system (SAMP3). For these measurements, approximately 100 mg of the sample were deposited in quartz wool, and the consumption of the gases was measured with a thermal conductivity detector (TCD). The particle size of the samples was determined using a Zetasizer Nano Series instrument (Malvern Panalytical, U.K.),

equipped with a 633 nm He-Ne laser. For the analysis, a suspension of the samples was prepared at a concentration of 0.1 g L^{-1}, using a 0.1 mmol L^{-1} $NaNO_3$ solution as the dispersing medium. The suspension was analyzed by dynamic light scattering (DLS), a technique that estimates the hydrodynamic diameter of particles in suspension based on fluctuations in the intensity of scattered light caused by the Brownian motion of the particles. The FTIR analysis was performed using pyridine as a probe molecule to investigate the nature and concentration of acid sites at room temperature. Pellets were prepared using potassium bromide (KBr) and accurately weighed samples, which were exposed to a pyridine atmosphere for 48 h in a vacuum desiccator. Infrared spectra were recorded in transmittance mode with 60 scans in the 4000-400 cm^{-1} region using a Shimadzu IRAffinity-T spectrophotometer. X-ray photoelectron spectroscopy (XPS) measurements were performed at the Electron Spectroscopy Laboratory (LEe-, UFRGS) using a SPECS system equipped with a PHOIBOS 150 1D-DLD hemispherical electron analyzer. An Al Kα (1486.6 eV,150 W) X-ray source was employed. Survey spectra were acquired using a pass energy of 50 eV, an energy step size of 0.5 eV, and an acquisition time of 0.1 s. For high resolution spectra, the analyzer was set to a pass energy of 30 eV, a step size of 0.1 eV, and an acquisition time of 0.5 s. Energy calibration was performed using the Au $4f_{7/2}$peak obtained from a clean gold foil. An acceptance angle of ±5° was used, and the base pressure in the analysis chamber was 4×10^{-10} mbar. Spectra were analyzed using Casa XPS software [44], and charge correction was applied by referencing the adventitious C 1s peak (C–C/C–H) to a binding energy of 284.8 eV [45]. The peak fitting procedure accounted for line shape asymmetry using a30% Gaussian-Lorentzian ratio and a Shirley-type background [46]. The energy dispersive X-ray spectroscopy (EDX) analyses were performed using a Shimadzu EDX-720 spectrometer equipped with a source of rhodium radiation, operating at 15 kV (Na-Sc) or 50 kV (Ti-U) and a 5 mm collimating slit in air.

The nature and concentration of the acid sites were evaluated by FTIR spectroscopy of adsorbed pyridine. The concentrations of Brønsted and Lewis acid sites were calculated according to the procedure described by Dos Santos et al. (2021), using Equation 4:

$$q_{B,L} = \frac{A_{B,L}\pi D^2}{4wE_{B,L}}$$

where $q_{B,L}$ is the concentration of Brønsted or Lewis acid sites, $A_{B,L}$ is the integrated area of the corresponding absorption band, D is the pellet diameter, w is the mass of the sample in the pellet, and $E_{B,L}$ is the extinction coefficient of pyridine adsorbed on the corresponding acid site. The bands in the ranges of 1435–1445 and 1580–1588 cm^{-1} were integrated for Lewis and Brønsted acid sites, respectively. Extinction coefficients of 2.22 ± 0.21cm μmol^{-1} for Lewis sites and 1.67 ± 0.12cm μmol^{-1} for Brønsted sites were employed. The B/L ratio was calculated as the ratio between the corresponding acid-site concentrations, q_B/q_L.

Catalytic tests of fructose conversion

Catalytic tests of fructose conversion were performed in glass vials under magnetic stirring at 160 °C, with reaction times ranging from 1 to 6 h. The experimental parameters consisted of 0.016 g of fructose, 2.0 mL of deionized water, and 0.0030 g of catalyst (Table 1), when applicable, in each glass vial.

At the end of each reaction, the reaction mixture was filtered through 0.45 µm membrane filters (Millipore™). The resulting solutions were analyzed by high-performance liquid chromatography (HPLC) with refractive index (RI) detection using a SHIMADZU (Japan) CTO-20A system equipped with a Varian MetaCarb H Plus (300 x 7.8 mm) column and a Varian MetaCarb H Plus guard column and the operating parameters are provided in the Supplementary Information. Calibration curves prepared from standard solutions were used for product quantification and fructose conversion, as well as product selectivity and yield, were determined as described below.

Conversion was determined according to Eq. (1), based on the initial fructose concentration (C_o) and the final concentration (C_f) in mol L^{-1}.

$$C(\%) = \left(\frac{C_0 - C_f}{C_0}\right) x\ 100 (1)$$

The yield (Y) of each identified soluble product was calculated according to Eq. (2), where C_i represents the concentration of product i (mol L^{-1}) and C_0corresponds to the initial fructose concentration (mol L^{-1}).

$$Y_i(\%) = \left(\frac{C_i}{C_0}\right) x 100 (2)$$

The selectivity (S) of each product was calculated according to Eq. (3), where $C_i$represents the concentration of product i (mol L^{-1}) and C_{i1},C_{i2},C_{i3},C_{i4},C_{i5}correspond to the concentrations of the remaining products (mol L^{-1}).

$$S_i(\%) = \left(\frac{C_i}{C_i + C_{i2} + C_{i3} + C_{i4} + C_{i5}}\right) \quad (3)$$

Reusability tests were performed with CeNb25(P) at 160 °C for 2 h under the same reaction conditions described above. After each cycle, the catalyst was recovered by centrifugation and calcined at 550

°C for 4 h prior to reuse. This procedure was repeated successively until the end of the cycles. To evaluate possible leaching, the liquid samples collected after the reuse cycles were analyzed by energy-dispersive X-ray spectroscopy (EDX).

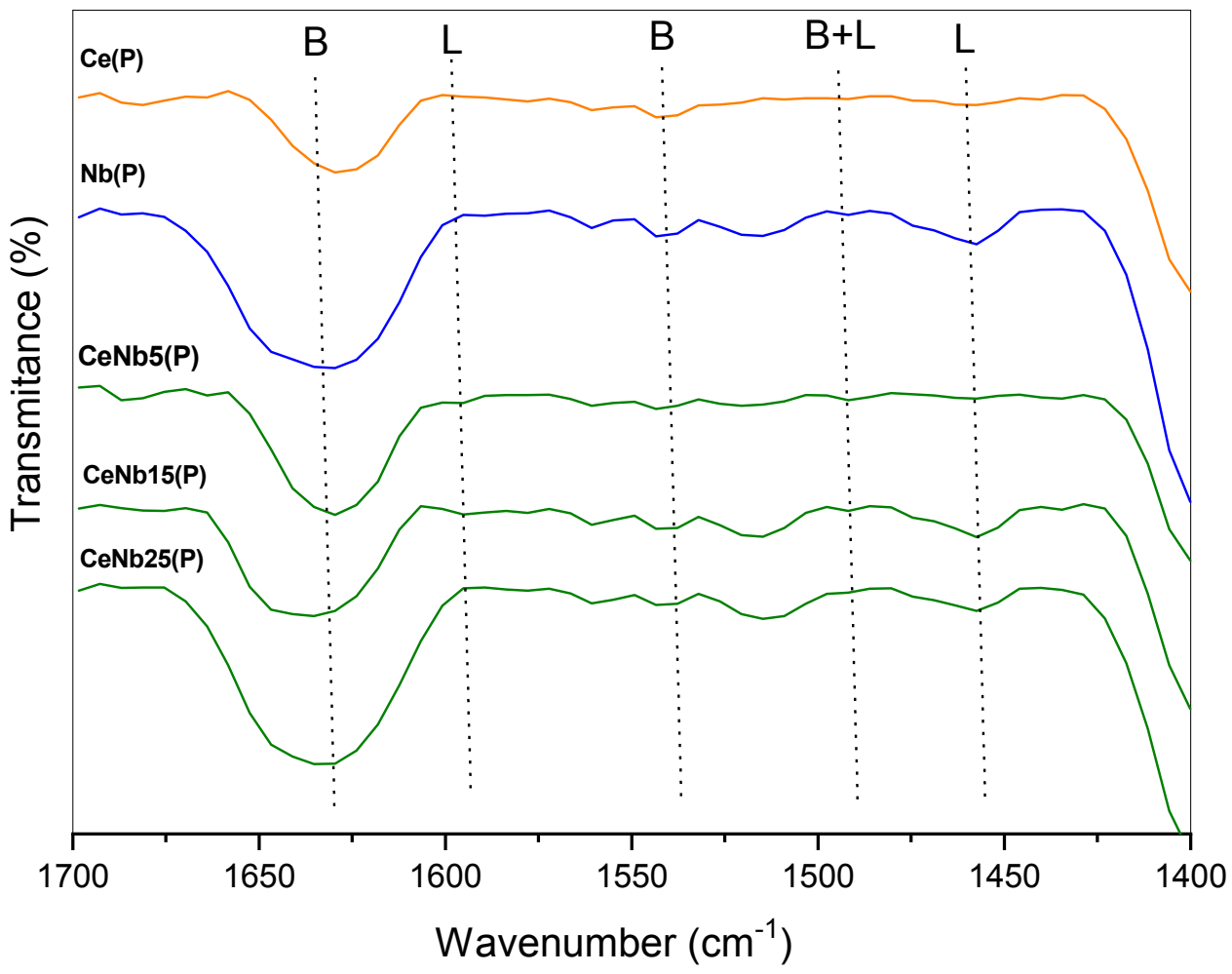

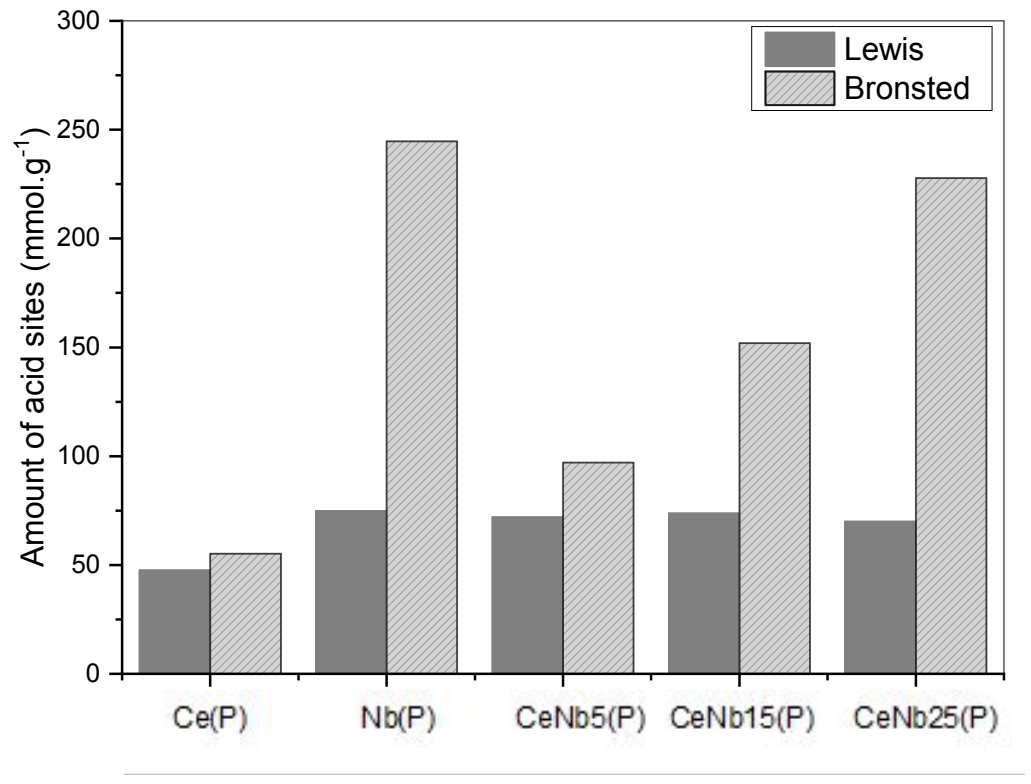

A **B**

Figure S1. Pyridine-FTIR spectra for Ce(P), Nb(P), CeNb5(P), CeNb15(P), and CeNb25(P), showing the bands assigned to Brønsted (B), Lewis (L), and combined Brønsted-Lewis (B + L) acid sites (**A**), and corresponding amounts of Brønsted and Lewis acid sites determined from the pyridine adsorption measurements (**B**).

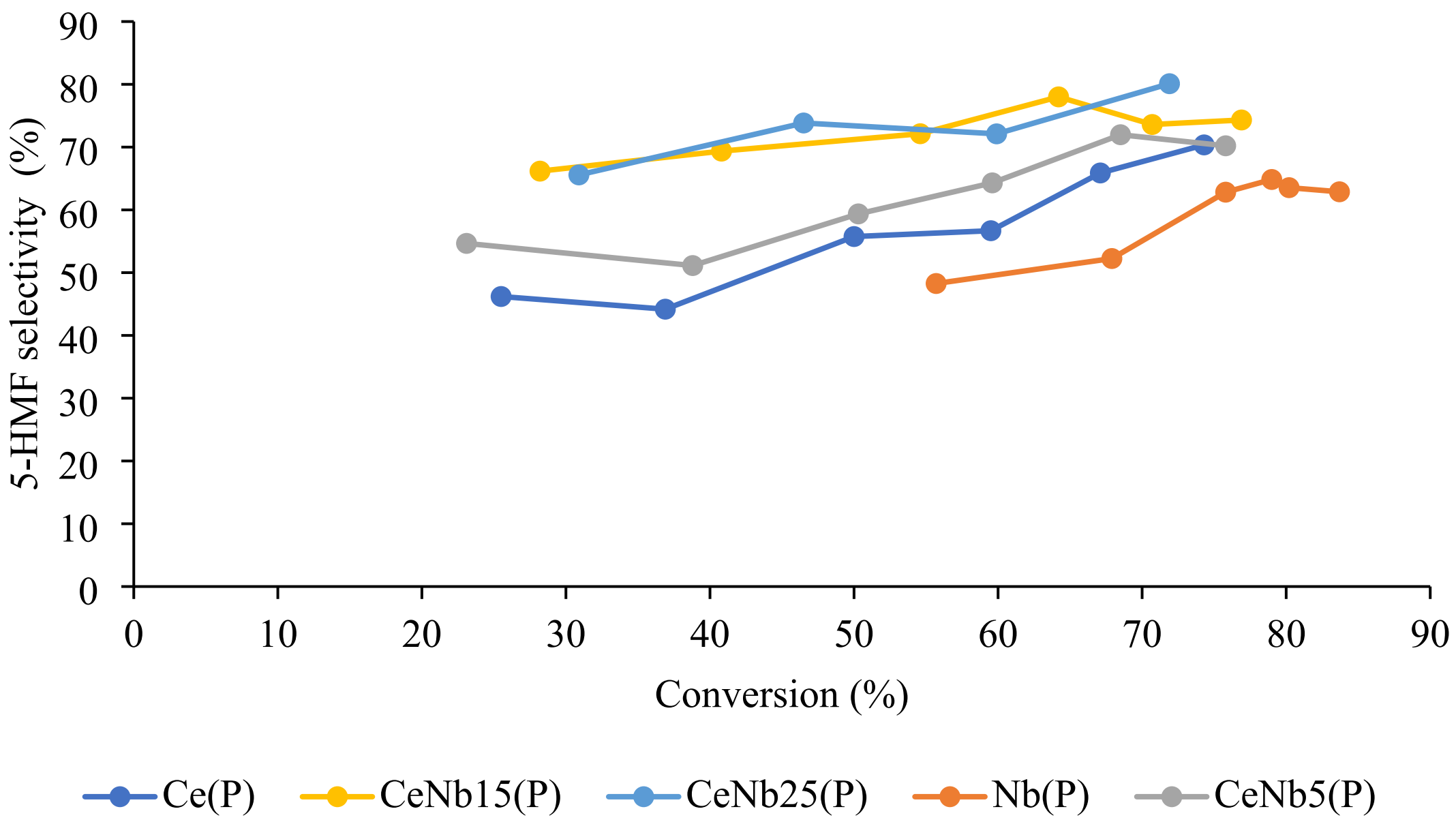

Figure S2. Selectivity of 5-HMF as a function of fructose conversion at 160 °C over Ce(P), Nb(P), CeNb5(P), CeNb15(P), and CeNb25(P); selectivity was calculated relative to the sum of the identified soluble products and the data points correspond to reaction times ranging from 1 to 6 h.

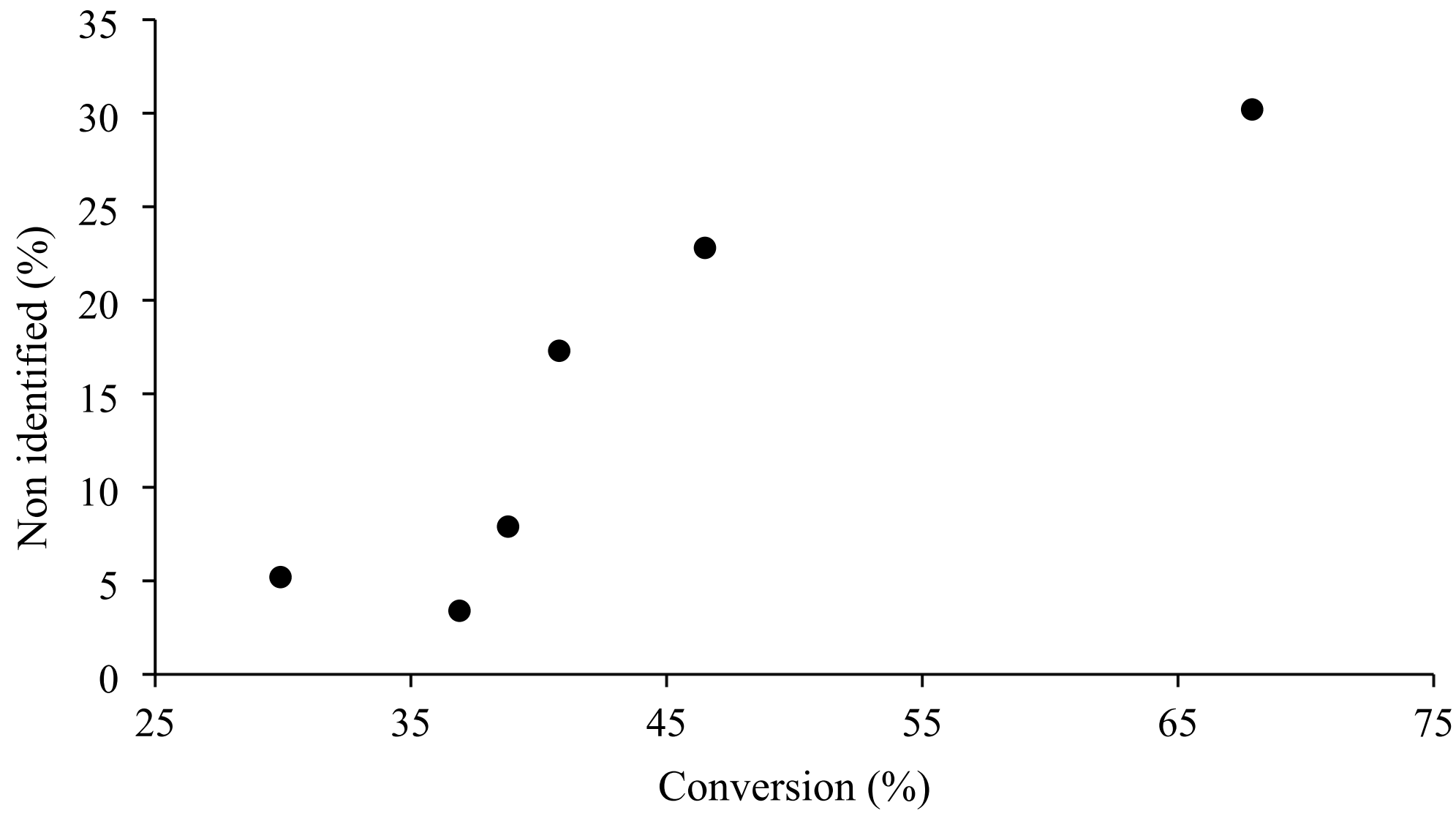

Figure S3. Relationship between fructose conversion and the unidentified fraction at 160 °C; the unidentified fraction was calculated as the difference between fructose conversion and the sum of the identified soluble product yields.

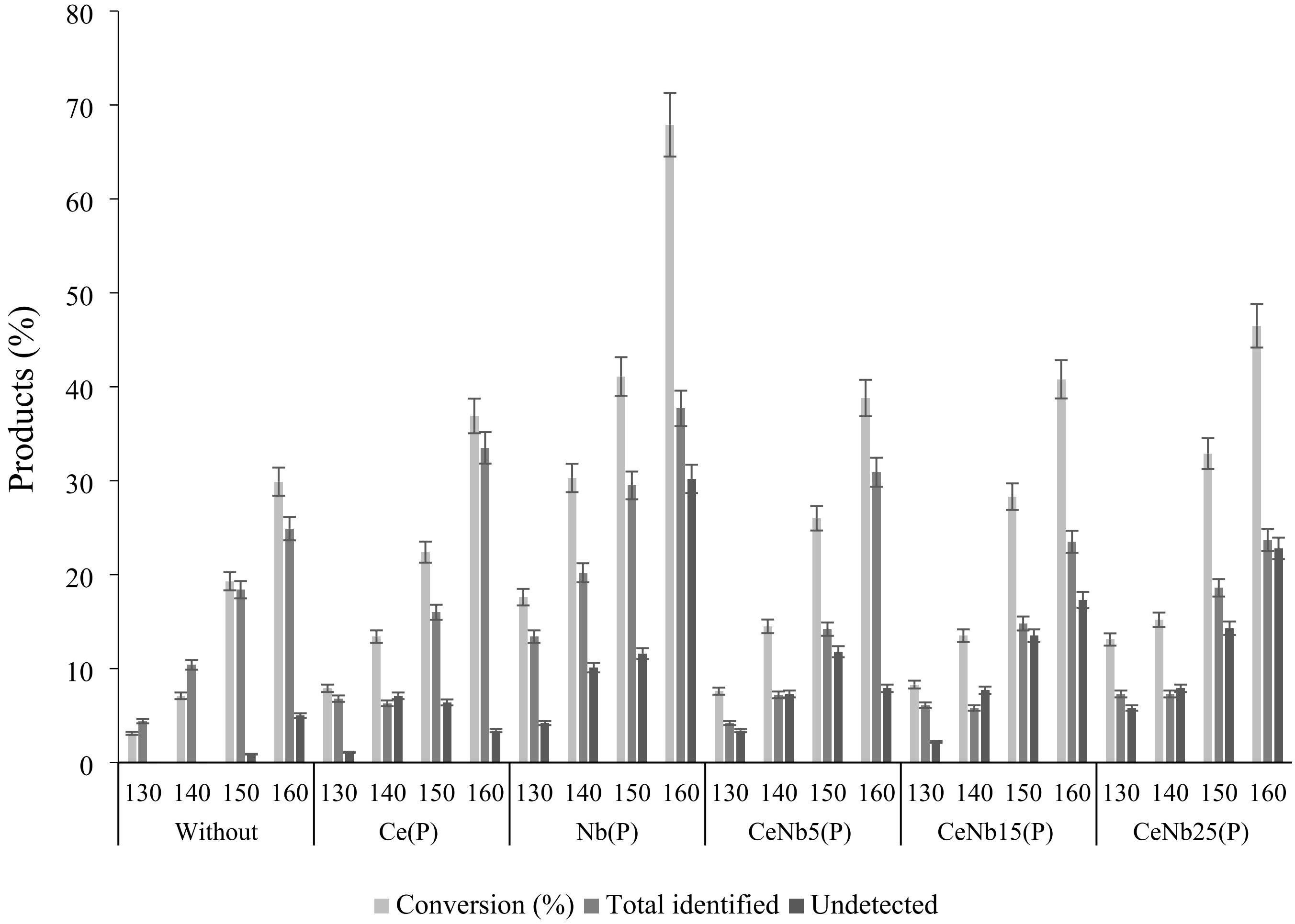

Figure S4. Comparison between fructose conversion (%) at 130, 140, 150 and 160 ºC, detected soluble products (%) and non identified fraction (%) for Ce-Nb catalysts (Non detected fraction (%) = Conversion (%) - soluble products identified (%)); error bars represent the estimated relative experimental uncertainty of ±5% associated with the chromatographic determinations.

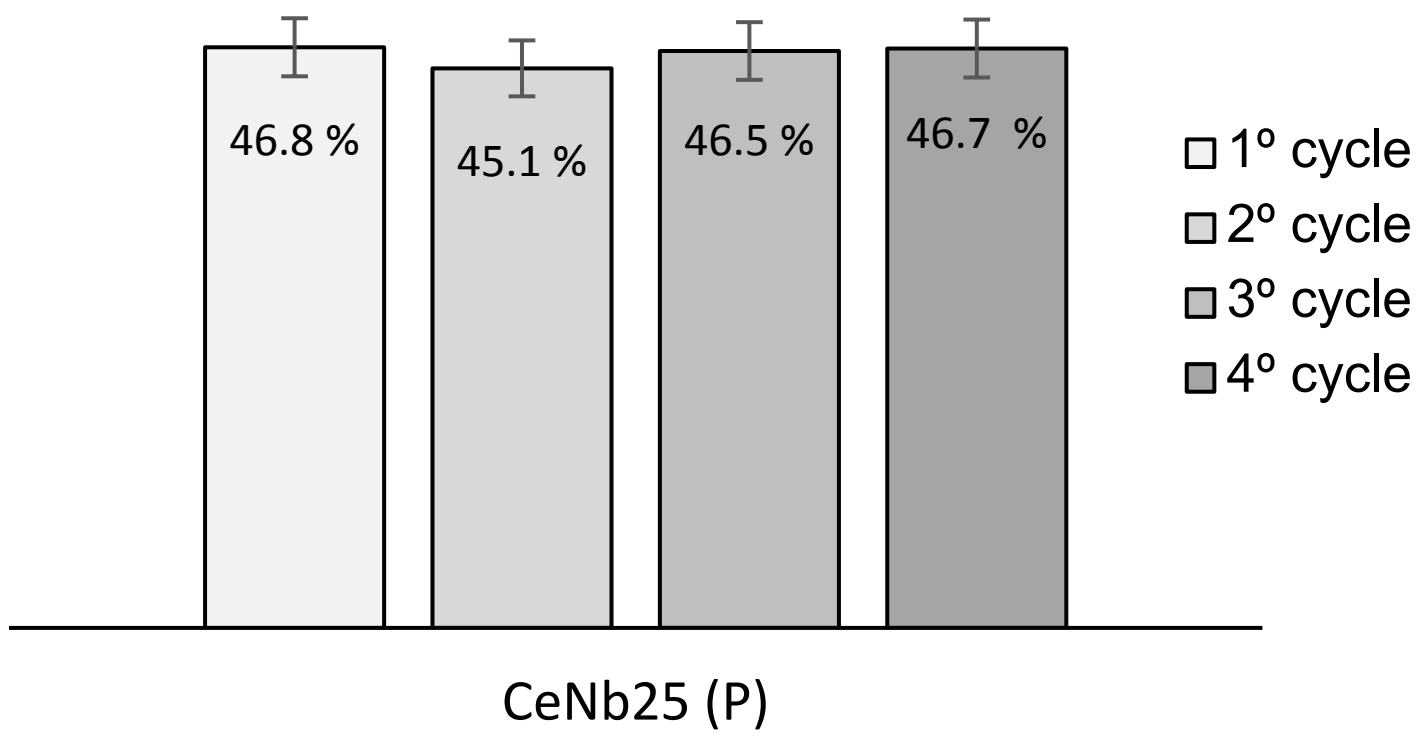

A

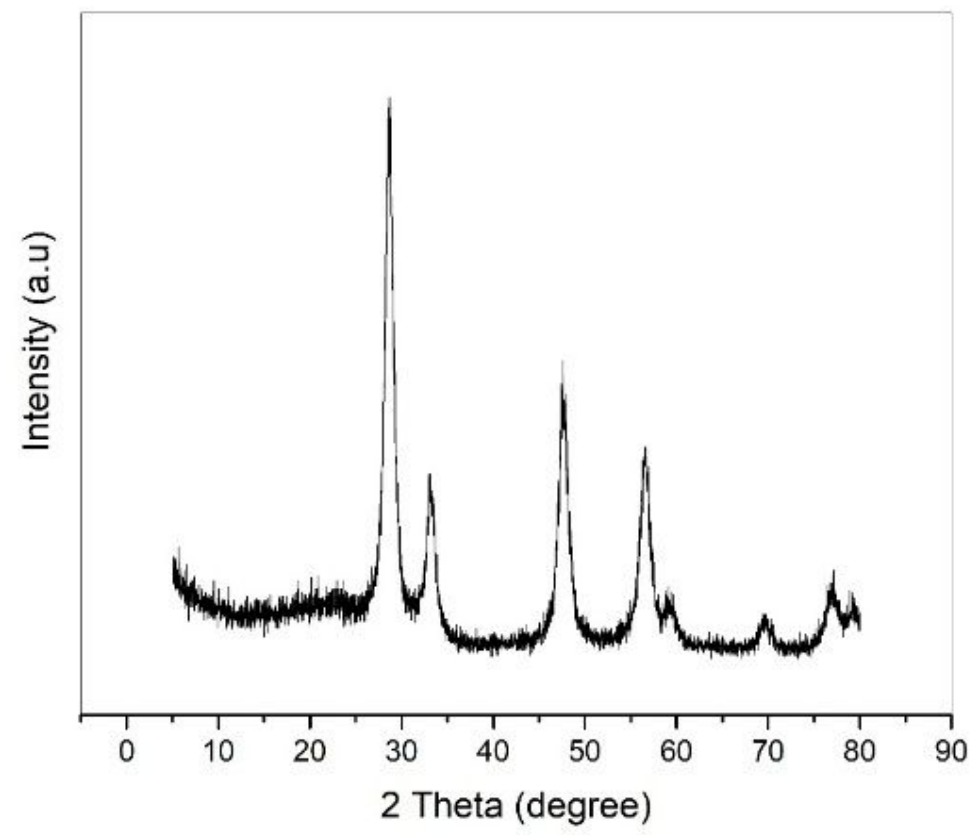

B

Figure S5. Conversion (%) of reuse test of CeNb25(P) at 160 °C for 2 h, four consecutive cycles; error bars represent the estimated relative experimental uncertainty of ±5% associated with the chromatographic determinations (**A**) and XRD pattern of CeNb25(P) after the reuse tests (**B**).

Table S1. Soluble products identified in the fructose conversion at 130 °C, 140 °C, 150 °C and 160 °C, 2 hours, without or using $3.0x10^{-3}$ g of Ce(P), Nb(P), CeNb5(P), CeNb15(P), and CeNb25(P) (Yield (%)); the estimated relative experimental uncertainty of the chromatographic determinations is ±5%

	G	5-HMF	GLY	PIRU	DHA	AL	ALE	AF	AA	**Conversion (%)**	**Total identified (%)**
Ce(P) 130 °C	1,1 ± 0,06	0,9 ± 0,05	0,3 ± 0,01	0,7 ± 0,03	0,0 ± 0,00	0,0 ± 0,00	0,0 ± 0,00	0,0 ± 0,00	0,0 ± 0,00	3,1 ± 0,16	3,0 ± 0,15
140 °C	1,9 ± 0,10	3,1 ± 0,16	0,0 ± 0,00	1,9 ± 0,10	0,0 ± 0,00	0,0 ± 0,00	0,0 ± 0,00	0,0 ± 0,00	0,0 ± 0,00	7,1 ± 0,35	6,9 ± 0,35
150 °C	2,9 ± 0,14	7,2 ± 0,36	0,7 ± 0,03	2,5 ± 0,12	0,3 ± 0,01	0,6 ± 0,03	0,1 ± 0,01	3,1 ± 0,16	1,0 ± 0,05	19,3 ± 0,97	18,5 ± 0,93
160 °C	1,6 ± 0,08	11,4 ± 0,57	0,8 ± 0,04	4,9 ± 0,25	0,3 ± 0,01	0,8 ± 0,04	0,1 ± 0,01	3,8 ± 0,19	1,2 ± 0,06	29,9 ± 1,50	24,7 ± 1,24
Nb(P) 130 °C	2,2 ± 0,11	1,1 ± 0,06	1,1 ± 0,06	1,5 ± 0,08	0,0 ± 0,00	0,0 ± 0,00	0,0 ± 0,00	0,9 ± 0,05	0,0 ± 0,00	7,9 ± 0,40	6,8 ± 0,34
140 °C	1,3 ± 0,07	2,3 ± 0,11	0,5 ± 0,03	1,2 ± 0,06	0,0 ± 0,00	0,0 ± 0,00	0,0 ± 0,00	1,0 ± 0,05	0,0 ± 0,00	13,4 ± 0,67	6,3 ± 0,32
150 °C	1,3 ± 0,07	7,0 ± 0,35	0,9 ± 0,05	2,2 ± 0,11	0,3 ± 0,01	1,1 ± 0,06	0,0 ± 0,00	2,4 ± 0,12	0,8 ± 0,04	22,4 ± 1,12	16,0 ± 0,80
160 °C	1,8 ± 0,09	14,8 ± 0,74	1,8 ± 0,09	4,8 ± 0,24	0,6 ± 0,03	4,5 ± 0,23	0,1 ± 0,01	4,1 ± 0,20	1,5 ± 0,08	36,9 ± 1,84	33,5 ± 1,68
CeNb5(P) 130 °C	1,2 ± 0,06	4,8 ± 0,24	1,2 ± 0,06	2,6 ± 0,13	0,6 ± 0,03	1,9 ± 0,10	0,0 ± 0,00	1,1 ± 0,06	0,0 ± 0,00	17,6 ± 0,88	13,4 ± 0,67
140 °C	1,5 ± 0,08	8,2 ± 0,41	0,5 ± 0,03	3,8 ± 0,19	0,8 ± 0,04	3,5 ± 0,18	0,0 ± 0,00	1,9 ± 0,10	0,0 ± 0,00	30,3 ± 1,52	20,2 ± 1,01
150 °C	1,6 ± 0,08	14,2 ± 0,71	0,6 ± 0,03	3,6 ± 0,18	0,5 ± 0,03	4,3 ± 0,21	0,0 ± 0,00	3,1 ± 0,16	1,4 ± 0,07	41,1 ± 2,06	29,5 ± 1,48
160 °C	0,9 ± 0,05	19,7 ± 0,98	0,6 ± 0,03	2,0 ± 0,10	0,0 ± 0,00	5,2 ± 0,26	0,4 ± 0,02	3,1 ± 0,16	5,7 ± 0,29	67,9 ± 3,40	37,7 ± 1,89
CeNb15(P) 130 °C	1,9 ± 0,10	0,7 ± 0,03	0,3 ± 0,01	0,7 ± 0,03	0,0 ± 0,00	0,0 ± 0,00	0,0 ± 0,00	0,6 ± 0,03	0,0 ± 0,00	7,6 ± 0,38	4,2 ± 0,21
140 °C	1,8 ± 0,09	1,9 ± 0,10	0,6 ± 0,03	1,4 ± 0,07	0,0 ± 0,00	0,0 ± 0,00	0,0 ± 0,00	1,5 ± 0,08	0,0 ± 0,00	14,5 ± 0,73	7,2 ± 0,36
150 °C	0,7 ± 0,03	7,4 ± 0,37	0,7 ± 0,03	0,1 ± 0,01	0,4 ± 0,02	1,5 ± 0,08	0,0 ± 0,00	3,1 ± 0,16	1,4 ± 0,07	26,0 ± 1,30	14,2 ± 0,71
160 °C	1,6 ± 0,08	15,8 ± 0,79	1,1 ± 0,06	3,9 ± 0,20	0,5 ± 0,03	3,5 ± 0,18	0,0 ± 0,00	3,8 ± 0,19	1,2 ± 0,06	38,8 ± 1,94	30,9 ± 1,54
CeNb25(P) 130 °C	2,1 ± 0,11	1,3 ± 0,07	1,0 ± 0,05	1,1 ± 0,06	0,0 ± 0,00	0,0 ± 0,00	0,0 ± 0,00	0,7 ± 0,03	0,0 ± 0,00	8,3 ± 0,42	6,1 ± 0,30
140 °C	1,4 ± 0,07	2,2 ± 0,11	0,5 ± 0,03	0,9 ± 0,05	0,0 ± 0,00	0,0 ± 0,00	0,0 ± 0,00	0,7 ± 0,03	0,0 ± 0,00	13,5 ± 0,68	5,8 ± 0,29
150 °C	1,6 ± 0,08	7,0 ± 0,35	0,7 ± 0,03	2,3 ± 0,11	0,0 ± 0,00	0,6 ± 0,03	0,0 ± 0,00	2,3 ± 0,11	0,3 ± 0,01	28,3 ± 1,42	14,8 ± 0,74
160 °C	0,6 ± 0,03	16,3 ± 0,82	0,5 ± 0,03	2,1 ± 0,11	1,1 ± 0,06	1,5 ± 0,08	0,2 ± 0,01	1,6 ± 0,08	0,7 ± 0,03	40,8 ± 2,04	23,5 ± 1,18

5-HMF= 5-(hydroxymethyl)furfural; G= glucose; GLY= glyceraldehyde; Piru=pyruvaldehyde; AA= aceticacid; ALE= levulinicacid; AF= formicacid; AL= lacticacid; DHA= dihydroxycetone.
